# Pharmacokinetics and Tissue Distribution Kinetics of Puerarin in Rats Using Indirect Competitive ELISA

**DOI:** 10.3390/molecules22060939

**Published:** 2017-06-05

**Authors:** Hui Kong, Xueqian Wang, Rongfeng Shi, Yan Zhao, Jinjun Cheng, Xin Yan, Xiaoman Liu, Yongzhi Wang, Meiling Zhang, Qingguo Wang, Huihua Qu

**Affiliations:** 1 School of Chinese Medicine, Beijing Key Laboratory, Beijing University of Chinese Medicine, Beijing 100029, China; doris7629@126.com (H.K.); shirlyding@163.com (X.W.); srflab@sina.com (R.S.); zhaoyandr@gmail.com (Y.Z.); carlosjjcheng@163.com (J.C.); liuxiaoman890509@163.com (X.L.); wangqg8558@sina.com (Q.W.); 2 School of Chinese Materia Medica, Beijing University of Chinese Medicine, Beijing 100029, China; 20150931805@bucm.edu.cn (X.Y.); 20150931804@bucm.edu.cn (Y.W.); 18811790361@163.com (M.Z.); 3 Beijing Institute of Traditional Chinese Medicine, Beijing University of Chinese Medicine, Beijing 100029, China

**Keywords:** pharmacokinetics, tissue distribution, indirect competitive enzyme-linked immunosorbent assay, puerarin

## Abstract

Puerarin (PUE) is a compound isolated from the roots of *Pueraria lobata*. We studied the pharmacokinetics and tissue distribution kinetics of PUE in Sprague-Dawley rats following intraperitoneal administration of three concentrations. Indirect competitive ELISA based on an anti-PUE monoclonal antibody was used to determine the concentration of PUE in the blood, heart, liver, spleen, lung, kidney, hippocampus, cerebral cortex, and striatum. The plasma and tissue distribution kinetic characteristics following a single injection of PUE (20, 40 and 80 mg/kg) were calculated using a non-compartment model. In the high-dose (80 mg/kg) and medium-dose (40 mg/kg) groups, the kinetic profile of PUE in blood and kidney samples showed two absorption peaks, while that of the other tissues showed only one peak. In the low-dose (20 mg/kg) group, there was only one peak, irrespective of the sample type. Pharmacokinetic parameters, such as the area under the curve, C_max_, and T_max_ varied according to the administered dose. AUC and C_max_ values increased dose-dependently. PUE was widely distributed in areas of the brain such as the hippocampus, cerebral cortex, and striatum, providing a foundation for guiding the use of PUE in the treatment of cerebral ischaemic stroke and neurodegenerative diseases.

## 1. Introduction

**Figure 1:**
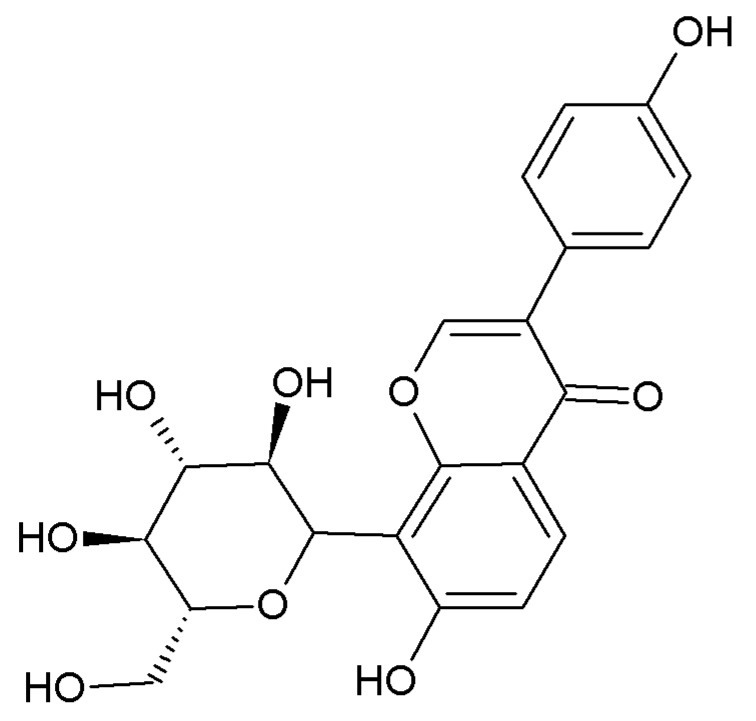
Chemical structure of puerarin.

Puerarin (PUE, Figure 1) is a major bioactive compound isolated from the dried roots of *Pueraria lobata* (Willd.) Ohwi (*Fabaceae*) [1]. Clinically, PUE has been widely used to treat ischaemic stroke in China [2]. It acts by dilating blood vessels, improving cerebral (pial) microcirculation and blood flow [3], reducing blood viscosity, platelet aggregation, and microintravascular fibrin precipitation, inhibiting thrombus formation [4], and attenuating neuronal apoptosis [5]. Modern pharmacological research has shown that PUE inhibits inflammatory reactions [6], possesses antithrombotic [7], and hypoglycaemic [8] properties, and suppresses oxidative stress [9].

The study of drug pharmacokinetics and distribution is one of the key steps in elucidating the mechanism of drug action. Thus far, high-performance liquid chromatography (HPLC) [6], liquid chromatography coupled with tandem mass spectrometry (LC-MS/MS) [10], gas chromatography [11], and gas chromatography-mass spectrometry [12] are the most widely used techniques in pharmacokinetic studies. However, sample pretreatment for these methods is complex, requiring the removal of proteins and a concentration step, which inevitably leads to sample loss. This is especially problematic when the sample concentration is relatively low and the loss rate is high. Hence, the drug concentration detected is not the actual concentration. Compared with the processing of blood samples, tissue processing is more complex and the drug content therein is much lower. Thus, it is more difficult to enrich drugs to detectable concentrations.

Studying the tissue distribution of drugs is complicated by the above reasons. Whole-organism drug distribution studies at a single time point after a single-dose administration are common, but drug kinetics in specific organs are given less attention. Questions related to how the drug distribution varies in organs and whether different drug doses lead to changes in distribution remain difficult to answer owing to the limitations of current detection methods. To address these questions it is, therefore, necessary to develop new techniques for monitoring tissue drug distribution.

It has been shown that PUE can cross the blood-brain barrier [13], but there are no reports on its differential distribution in the regions of the brain. PUE has been reported to significantly dilate coronary arteries, protect neurons from damage by glutamine and *N*-methyl-d-aspartate, and improve microcirculation in both animals and patients with cardiovascular and cerebral vascular diseases, especially ischaemic dementia [14]. However, how PUE is distributed in the brain regions related to cognitive functions, such as the hippocampus, whether it can reach the effective concentration, and how long it can remain in the target regions is unclear.

In our previous study, we developed an indirect competitive enzyme-linked immunosorbent assay (icELISA) based on monoclonal antibodies (MAbs), and successfully applied them to detect PUE in biological samples such as blood [15] and saliva [16]. This method offers advantages in pharmacokinetic study, including simple sample preparation requiring only phosphate-buffered saline (PBS), water, or other solvents for dilution [17], high sensitivity to detect trace amounts of sample [18], high specificity, easy operation, and high-throughput capability to detect a large number of samples simultaneously [19,20].

In this study, we applied the method to characterise the pharmacokinetics and tissue distribution kinetics of PUE. The aim of the present study was to determine the distribution of PUE in organs, especially the regularity of change in PUE concentration over time in the hippocampus, cerebral cortex, and striatum, and to observe the effects of various doses on its distribution. The kinetic regularities of PUE in the blood, heart, liver, spleen, lung, kidney, hippocampus, cerebral cortex, and striatum observed in this study may provide a mechanistic basis for treating stroke and neurodegenerative diseases.

## 2. Results and Discussion

**Figure 2:**
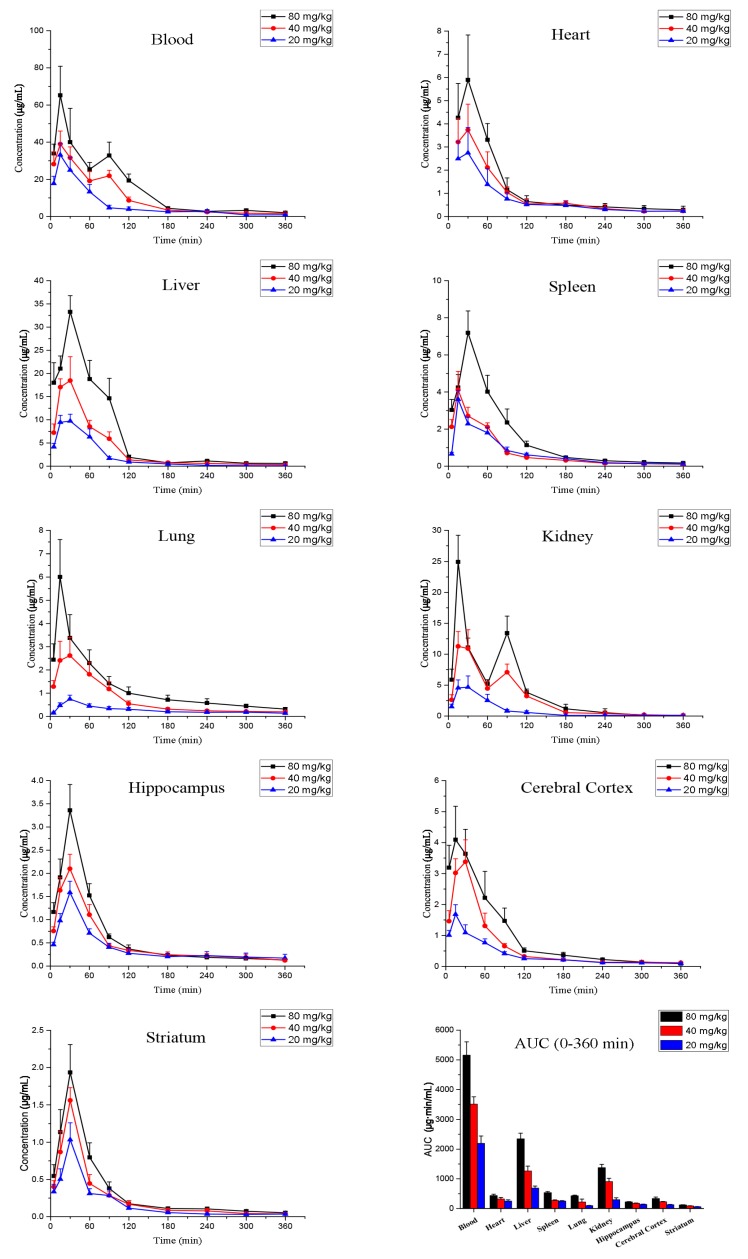
Mean concentration-time profiles at three different concentrations of puerarin following intraperitoneal injection (mean ± SD, *n* = 6). AUC, area under the concentration-time curve.

icELISA was used to analyse samples of rat blood, heart, liver, spleen, lung, kidney, hippocampus, cerebral cortex, and striatum. Equations for the calibration curves of PUE in blank samples are shown in Table 1. The main pharmacokinetic data are summarised in Table 2. The mean concentration vs. time profiles at three different PUE concentrations are presented in Figure 2. Pharmacokinetic data from other published studies are given in Table 3. The mean PUE concentrations in blood and various tissues at different time points following PUE injection are presented in Table 4.

In our previously study [16], the cross reactivity of the anti-PUE Mab was checked, except for 58.10% cross-reactivity against baicalin and 48.2% cross-reactivity against daidzin. The cross-reactivity against any of the other related compounds was <0.01%. Because baicalin was not administered in current study, baicalin had no impact on the PUE determination. The 48.2% cross-reactivity against daidzin was detected alone, without PUE in the reaction. Since PUE and daidzin were coexist in pueraria granules, we analysed the PUE content in saliva samples after administration of pueraria granules by both icELISA and HPLC, and found almost identical results. Therefore, daidzin has little effect on PUE determination by icELISA when both compounds are present.

Unmodified PUE has been reported to be a major component in blood and urine, indicating that phase II metabolism is not the major metabolic pathway for PUE excretion [21], and two glucuronidated metabolites of PUE (puerarin-7-*O*-glucuronide and puerarin-4′-*O*-glucuronide) were the main metabolites in plasma samples identified by rapid resolution liquid chromatography electrospray ionization collision induced dissociation tandem mass spectrometry (RRLC-ESI-CID–MS/MS) following intravenously administration to rats [22].

The icELISA method can’t intuitively reflect the tested compound formula, which is a limitation. In this study, the metabolites and PUE were present in the blood and tissues. Puerarin-7-*O*-glucuronide and puerarin-4′-*O*-glucuronide are all structural analogues, and may have cross-reactivity against the Mab when analysed alone. However, as mentioned above for daidzin, the metabolites’ influence on the determination of PUE content would be very small.

Table 3, shows a wide range in AUC values, which differ from our findings. This discrepancy may derive from the differences in analytical methods and sample processing. In our study, the plasma samples were diluted only with PBS prior to icELISA analysis, but serum proteins must be precipitated with methanol or other organic solvents before HPLC or HPLC-MS, increasing the risk of PUE loss. Additionally, the dose route, the time of the last data point and the steep slope of the curve at the beginning also account for the differences.

Wu et al. [25] reported that PUE concentrations were much higher in the liver and lung than in the kidney, spleen, uterus, heart, and other organs in mice 60 min following oral administration of 200 mg/kg. However, they found that PUE concentration was highest in the uterus and muscle, and lower in the kidney, liver, heart, lung, and brain 60 min after intravenous injection. Conversely, Cheng et al. [26] reported that PUE was abundantly distributed in the kidney, heart, and liver, compared with its distribution in the brain, lung, and spleen of rats 60 min after oral administration of a Shange lipid-lowering dispersible tablets solution containing 175.6 mg/kg PUE. Prasain et al. [21] reported that the highest PUE concentration in rats was found in the lungs, followed by kidney, pancreas, and liver 120 min after oral administration of 50 mg/kg. This was similar to the descending order of distribution found by Li et al. [27] after oral administration of 400 mg/kg PUE. From Table 4, we monitored the distribution of PUE in the blood, heart, liver, spleen, lung, kidney, cerebral cortex, hippocampus, and striatum at 10 different time points after intraperitoneal injection of 80, 40 and 20 mg/kg (Table 4), constituting a relatively complete kinetic study. In all rat experimental groups, the AUC_0–360 min_ values indicated that PUE was mainly distributed in the liver and kidney, and that its concentration in the lung was relatively low. It is evident from the coparisions that the formulations, dose, and dose route and time of administration all affect the distribution of PUE in various tissues. In particular, PUE distribution in the lung is relatively low when administered by injection, but high following oral treatment.

In the 40-mg/kg and 80-mg/kg groups, the kinetics of PUE in the blood and kidney showed two peaks (Figure 2), which may indicate reabsorption of the molecule. In contrast, there was only one absorption peak in the other tissues. Interestingly, in the 20-mg/kg group (low-dose group), there was only one peak in the PUE metabolic profile, irrespective of the sample type. Li et al. reported an absorption peak approximately 0.89 h following oral administration of 400 mg/kg in rats [27]. Jung et al. reported one peak in human blood 1.18 h after oral administration of Gegen extract, which contained 9.984 mg PUE [28]. However, in human saliva, double peaks were seen approximately 49 min after oral administration of PUE at doses of 20, 40 and 60 mg/kg [16]. Double peaks in the concentration-time profile of PUE, whether in animal or human blood, may be attributable to enterohepatic recirculation at high doses, as was reported by Prasain et al. [21]. However, only one absorption peak has been observed at low doses, perhaps because enterohepatic recirculation is relatively weak at those levels.

PUE rapidly distributed into organs and crossed the blood-brain barrier to reach the hippocampus and striatum approximately 30 min after intraperitoneal administration (Figure 2). This provides a basis for its therapeutic potential against ischaemic brain damage. Nevertheless, it should be noted that the distribution of PUE into the different organs varied according to the administered dose. PUE quickly reached C_max_ in the spleen in the 20 and 40 mg/kg groups. In the 80 mg/kg group (high-dose group), however, the T_max_ in the spleen was twice as long.

In our previous study [29], the pharmacokinetics of PUE administered orally in mice at three different concentrations (100, 200 and 300 mg/kg) showed nonlinear characteristics, probably because PUE absorption has an inhibitory effect on its concentration [30]. We showed here that the AUC_0–360 min_ in blood and various tissues increased as the dose increased, indicating that PUE may exert a dose-dependent effect in the range of 20–80 mg/kg. These data may be useful in guiding future clinical applications of PUE.

## 3. Materials and Methods

### 3.1. Chemicals and Reagents

PUE (production batch number: 150303) was purchased from Guangzhou Baiyun Mountain Tianxin Pharmaceutical Limited Company (Guangzhou, China). The coating antigen PUE-bovine serum albumin (PUE-BSA) and anti-PUE-MAb from ascitic fluid were produced in our lab as described previously [15]. Goat anti-mouse immunoglobulin conjugated to horseradish peroxidase (GaMIgG-HRP, whole molecule) was purchased from GE Healthcare (Little Chalfont, UK). Ninety-six-well immunoplates were purchased from Corning Inc. (Corning, NY, USA). Skim milk was purchased from Becton Dickinson (Franklin Lakes, NJ, USA). 3,3′,5,5′-Tetramethylbenzidine (TMB) was purchased from Sigma-Aldrich (St. Louis, MO, USA). All other commercial chemicals were of analytical grade (AR) and were obtained from Sinopharm Chemical Reagents Beijing Co., Ltd. (Beijing, China).

### 3.2. Instruments

A spectrophotometric microtitre reader (Multiskan MK3; Thermo Fisher Scientific, Waltham, MA, USA) was used for absorbance measurements. An electro-heating standing temperature cultivator (DRP-9082) was purchased from Samsung Laboratory Instrument Co., Ltd. Shanghai, China. A high-speed refrigerated centrifuge (HC-2518R) was purchased from Anhui USTC Zonkia Scientific Instruments Co., Ltd. Anhui, China. An MM400 mixer-type grinding apparatus was purchased from Frederick Instrument and Equipment Co., Ltd., Shanghai, China.

### 3.3. Animals and Drug Administration

The study was performed according to the Guide for the Care and Use of Laboratory Animals approved by the Ethics Committee of Animal Experimentation of Beijing University of Chinese Medicine (approval no. 2013 BZHYLL00106). One-hundred eighty-one Sprague-Dawley rats (certificate of quality no. 11401500007163) weighing 220 ± 10 g were purchased from Sibeifu Experimental Animal Science and Technology Co., Ltd. (Beijing, China). The rats were kept in an environmentally-controlled breeding room for one week before the experiments, and had free access to standard laboratory chow and water. The animals were fasted overnight before drug administration.

One rat was randomly selected as a blank control. The other 180 rats were randomly divided into three groups of 60, and intraperitoneally injected with different doses of PUE 20, 40 and 80 mg/kg. Rats in each group were further randomly divided into 10 subgroups of six for measurements at 10 time points.

Blood was collected from the abdominal aorta with a coagulation vacuum recovery vessel 5, 15, 30, 60, 90, 120, 180, 240, 300 and 360 min after PUE injection. The rats were injected with 10% chloral hydrate as anaesthesia before blood collection. After the blood was drawn, the rats were rapidly dissected. The heart was perfused with saline until the liver became white. Next, the heart, liver, spleen, lung, kidney, hippocampus, cerebral cortex, and striatum were excised, weighed, homogenised (1 mL PBS per 100 mg tissue sample, 30 Hz, 1.5 min), and centrifuged (4 °C, 11,995× *g*, 10 min) before isolating the supernatant. The blood was left to stand for 30 min, centrifuged (4 °C, 2082× *g*, 10 min), and the supernatant was collected. All supernatants were stored at −20 °C prior to analysis. The stored supernatants were thawed at room temperature and diluted 20-, 50- or 100-fold with PBS to meet the concentration range of the calibration curve. Processed blood, heart, liver, spleen, lung, kidney, hippocampus, cerebral cortex, and striatum samples from the untreated rat were diluted similarly with PBS and used as controls.

### 3.4. icELISA

The icELISA procedure to measure PUE was performed as described previously [15,16]. Briefly, the coating antigen PUE-BSA (0.1 μg/mL) was added to a microtitre plate and incubated. The plates were rinsed three times. A 50-μL sample and an equal volume of ascitic fluid containing anti-PUE-MAb (1:10,000) were added to the wells, and the mixture was incubated for 1 h. After rinsing three times, the antibody was reacted with 100 μL of GaMIgG-HRP (1:10,000 in PBS) for 30 min. The plates were rinsed, and 100 μL of the TMB substrate solution was added to each well. After 15 min of incubation, the reaction was stopped by the addition of 50 μL of a stop solution (2 M H_2_SO_4_). The absorbance was measured at 450 nm (A_450_) using a spectrophotometric microplate reader.

### 3.5. Calibration Curve

Stock solutions of PUE (1 mg/mL) were prepared in a PBS/methanol mixture (80:20) and then diluted with PBS to a concentration of 1 μg/mL. The PUE stock solution (780 μL) was mixed with blank rat plasma (diluted 100-fold with PBS) in a centrifuge tube to a final volume of 1 mL. The mixture underwent a two-fold serial dilution with blank rat plasma so that the resulting plasma contained 780.00, 390.00, 195.00, 97.50, 48.75, 24.38, 12.19, 6.09 and 3.05 ng/mL PUE. The plasma was then processed according to the icELISA procedure above. The calibration curve was obtained by plotting A_450_ against ln C_PUE_. The limit of quantification was defined as the lowest concentration determined from the calibration curve. The standard curves for PUE concentration in the heart, liver, spleen, lung, kidney, hippocampus, cerebral cortex, and striatum were obtained in a similar manner.

### 3.6. Pharmacokinetics

Pharmacokinetic parameters such as the maximum concentration of PUE in the blood (C_max_), the time taken to reach the maximum concentration (T_max_), AUC_0–t_, and the mean residence time (MRT) were calculated using a non-compartment model with Kinetica software (version 5.0; Kinetica, Arlington, VA, USA). All other results are expressed as mean ± standard deviation.

## 4. Conclusions

PUE was widely distributed in areas of the brain such as the hippocampus, cerebral cortex, and striatum, providing a foundation for guiding the use of PUE in the treatment of cerebral ischaemic stroke and neurodegenerative diseases.

## Figures and Tables

**Table 1:** Calibration curves of puerarin in blank samples.

Sample	Regression Equation	*R*^2^	Detection Range (ng/mL)
Blood	Y = −0.19 ln (X) + 1.42	0.98	3.05–780
Heart	Y = −0.14 ln (X) + 1.05	0.97	3.05–780
Liver	Y = −0.14 ln (X) + 1.07	0.98	3.05–780
Spleen	Y = −0.14 ln (X) + 1.08	0.99	3.05–780
Lung	Y = −0.14 ln (X) + 1.04	0.97	3.05–780
Kidney	Y = −0.13 ln (X) + 1.01	0.95	3.05–780
Hippocampus	Y = −0.25 ln (X) + 1.89	0.97	3.05–780
Cerebral cortex	Y = −0.23 ln (X) + 1.90	0.97	3.05–780
Striatum	Y = −0.18 ln (X) + 1.23	0.96	3.05–780

**Table 2:** Pharmacokinetic parameters of puerarin in Sprague-Dawley rats following intraperitoneal administration (mean ± SD, *n* = 6).

Sample	Group (mg/kg)	AUC_0–360 min_ (µg·min/mL)	C_max_ (µg/mL)	T_max_ (min)	MRT (min)
	80	5157.76 ± 449.93	67.02 ± 12.87	18 ± 6	102 ± 14
Blood	40	3510.49 ± 244.72	40.10 ± 6.35	15 ± 0	95 ± 12
	20	2196.95 ± 246.74	33.66 ± 6.14	18 ± 6	94 ± 8
	80	438.32 ± 44.50	6.33 ± 1.56	28 ± 6	116 ± 16
Heart	40	323.64 ± 54.03	3.95 ± 0.74	25 ± 8	124 ± 11
	20	251.77 ± 44.14	2.95 ± 0.97	28 ± 6	167 ± 44
	80	2348.13 ± 191.24	33.27 ± 3.51	30 ± 0	76 ± 13
Liver	40	1264.43 ± 164.14	20.16 ± 1.85	28 ± 6	84 ± 20
	20	688.37 ± 68.26	10.91 ± 0.72	25 ± 8	63 ± 2
	80	536.07 ± 37.46	7.19 ± 1.18	30 ± 0	115 ± 30
Spleen	40	271.47 ± 20.66	4.11 ± 0.99	15 ± 0	97 ± 15
	20	252.19 ± 14.48	3.61 ± 0.48	15 ± 0	112 ± 12
	80	430.70 ± 21.97	5.81 ± 1.14	20 ± 8	146 ± 23
Lung	40	218.93 ± 98.66	3.08 ± 0.69	33 ± 15	148 ± 36
	20	97.75 ± 5.96	0.74 ± 0.16	25 ± 7	82 ± 40
	80	1377.37 ± 111.66	24.93 ± 4.26	15 ± 0	73 ± 9
Kidney	40	908.04 ± 109.56	11.63 ± 1.98	25 ± 8	75 ± 6
	20	299.92 ± 63.01	5.24 ± 0.93	25 ± 7	82 ± 40
	80	223.47 ± 9.21	3.35 ± 0.55	30 ± 0	123 ± 23
Hippocampus	40	175.19 ± 11.39	2.09 ± 0.31	30 ± 0	169 ± 38
	20	137.86 ± 9.98	1.58 ± 0.24	27 ± 6	163 ± 18
	80	336.88 ± 48.66	4.48 ± 0.86	22 ± 8	84 ± 7
Cerebral cortex	40	225.94 ± 9.95	3.56 ± 0.61	25 ± 8	116 ± 34
	20	130.32 ± 8.46	1.73 ± 0.24	17 ± 6	129 ± 20
	80	120.64 ± 8.72	1.93 ± 0.37	30 ± 0	103 ± 22
Striatum	40	88.09 ± 8.41	1.55 ± 0.17	30 ± 0	99 ± 17
	20	61.27 ± 7.86	1.03 ± 0.22	30 ± 0	113 ± 43

AUC, area under the concentration-time curve; C_max_, maximum concentration; T_max_, time at which maximum concentration is observed; MRT, mean residence time.

**Table 3:** Published pharmacokinetics of puerarin.

Sample	Dose (mg/kg)	Dose Route	AUC	Observation Period	Number of Data Points	Analysis Method	Literature Reference
Rat plasma	62.5	iv.	13.80 ± 1.71 μg·h/mL	0–240 min	9	HPLC	[13]
Rat plasma	32	iv.	2773.2 ± 572.3 ng·min/mL	0–240 min	8	HPLC	[14]
Rat serum	50	ig.	9.17 ± 4.87 mg·h/L	0–4 h	8	HPLC-MS/MS	[23]
Rat plasma	15	iv.	6587.04 ± 1520.60 ng·h/mL	0–8 h	9	LC-MS/MS	[24]
Rat plasma	80	ip.	5157.760 ± 449.934 µg·min/mL	0–360 min	10	ELISA	This paper
Rat plasma	40	ip.	3510.49 ± 244.72 µg·min/mL	0–360 min	10	ELISA	This paper
Rat plasma	20	ip.	2196.95 ± 246.74 µg·min/mL	0–360 min	10	ELISA	This paper

**Table 4:** Mean puerarin concentration (µg/mL) in blood and tissues at different time points following intraperitoneal injection (mean ± SD, *n* = 6).

Group (mg/kg)	Tissue	Time (min)									
		5	15	30	60	90	120	180	240	300	360
80	Blood	33.97 ± 4.94	65.26 ± 15.61	40.00 ± 18.22	25.38 ± 3.70	32.84 ± 7.15	19.36 ± 3.52	4.41 ± 0.67	2.80 ± 0.81	3.28 ± 0.72	2.05 ± 0.21
	Heart	1.96 ± 0.64	4.27 ± 1.47	5.89 ± 1.94	3.31 ± 0.70	1.16 ± 0.51	0.65 ± 0.25	0.49 ± 0.19	0.42 ± 0.15	0.34 ± 0.14	0.29 ± 0.16
	Liver	18.00 ± 4.32	21.04 ± 2.73	33.27 ± 3.51	18.79 ± 4.02	114.62 ± 4.33	1.93 ± 0.48	0.73 ± 0.11	1.14 ± 0.15	0.63 ± 0.06	0.62 ± 0.25
	Spleen	3.03 ± 0.57	4.24 ± 0.70	7.19 ± 1.18	4.02 ± 0.88	2.36 ± 0.73	1.13 ± 0.22	0.47 ± 0.06	0.30 ± 0.05	0.21 ± 0.02	0.17 ± 0.02
	Lung	2.43 ± 0.68	6.00 ± 1.60	3.38 ± 1.01	2.30 ± 0.57	1.42 ± 0.29	1.00 ± 0.27	0.71 ± 0.20	0.58 ± 0.18	0.44 ± 0.09	0.31 ± 0.07
	Kidney	5.87 ± 1.72	24.93 ± 4.27	11.09 ± 1.53	5.20 ± 0.69	13.42 ± 2.74	3.83 ± 0.54	1.18 ± 0.73	0.54 ± 0.63	0.14 ± 0.06	0.08 ± 0.03
	Hippocampus	1.17 ± 0.20	1.91 ± 0.40	3.36 ± 0.56	1.52 ± 0.25	0.62 ± 0.07	0.37 ± 0.09	0.23 ± 0.04	0.19 ± 0.04	0.16 ± 0.04	0.13 ± 0.02
	Cerebral cortex	3.19 ± 0.72	4.09 ± 1.08	3.64 ± 0.79	2.21 ± 0.86	1.47 ± 0.42	0.51 ± 0.09	0.36 ± 0.09	0.23 ± 0.03	0.14 ± 0.02	0.09 ± 0.04
	Striatum	0.55 ± 0.15	1.14 ± 0.30	1.93 ± 0.37	0.80 ± 0.19	0.38 ± 0.09	0.17 ± 0.04	0.11 ± 0.02	0.10 ± 0.03	0.07 ± 0.02	0.05 ± 0.02
40	Blood	28.21 ± 6.57	38.97 ± 7.06	31.63 ± 5.84	19.15 ± 5.02	21.92 ± 2.93	8.68 ± 1.78	3.43 ± 0.71	2.35 ± 0.23	1.73 ± 0.87	1.50 ± 0.34
	Heart	1.36 ± 0.27	3.22 ± 1.02	3.73 ± 1.11	2.11 ± 0.68	1.04 ± 0.24	0.54 ± 0.23	0.57 ± 0.11	0.34 ± 0.17	0.23 ± 0.04	0.24 ± 0.05
	Liver	7.23 ± 1.87	17.04 ± 1.79	18.46 ± 5.15	8.53 ± 1.36	5.92 ± 1.49	1.37 ± 0.13	0.75 ± 0.26	0.58 ± 0.12	0.43 ± 0.09	0.33 ± 0.09
	Spleen	2.12 ± 0.39	4.11 ± 0.99	2.71 ± 0.47	2.11 ± 0.23	0.71 ± 0.11	0.46 ± 0.05	0.31 ± 0.06	0.16 ± 0.03	0.14 ± 0.02	0.11 ± 0.02
	Lung	1.28 ± 0.26	2.41 ± 0.82	2.62 ± 0.78	1.81 ± 0.53	1.18 ± 0.31	0.54 ± 0.13	0.31 ± 0.06	0.24 ± 0.05	0.21 ± 0.05	0.20 ± 0.05
	Kidney	2.62 ± 0.83	11.28 ± 2.34	10.92 ± 3.02	4.45 ± 1.10	7.09 ± 1.31	3.22 ± 0.71	0.55 ± 0.27	0.40 ± 0.28	0.20 ± 0.07	0.11 ± 0.03
	Hippocampus	0.77 ± 0.09	1.63 ± 0.28	2.10 ± 0.31	1.11 ± 0.22	0.44 ± 0.05	0.34 ± 0.05	0.25 ± 0.06	0.22 ± 0.05	0.19 ± 0.07	0.12 ± 0.03
	Cerebral cortex	1.46 ± 0.34	3.02 ± 0.46	3.38 ± 0.71	1.31 ± 0.41	0.66 ± 0.09	0.32 ± 0.03	0.22 ± 0.04	0.13 ± 0.02	0.14 ± 0.04	0.12 ± 0.03
	Striatum	0.40 ± 0.08	0.87 ± 0.27	1.56 ± 0.17	0.44 ± 0.12	0.29 ± 0.05	0.17 ± 0.05	0.08 ± 0.03	0.08 ± 0.03	0.04 ± 0.01	0.04 ± 0.01
20	Blood	17.79 ± 3.82	33.19 ± 6.07	24.97 ± 6.88	13.32 ± 3.94	4.69 ± 1.36	3.89 ± 1.23	2.57 ± 0.31	2.73 ± 0.75	0.87 ± 0.18	0.89 ± 0.13
	Heart	1.05 ± 0.32	2.49 ± 0.74	2.75 ± 1.09	1.39 ± 0.68	0.76 ± 0.29	0.52 ± 0.12	0.48 ± 0.16	0.30 ± 0.07	0.23 ± 0.07	0.23 ± 0.04
	Liver	4.16 ± 0.76	9.45 ± 1.50	9.73 ± 1.48	6.32 ± 1.72	1.69 ± 0.42	0.89 ± 0.15	0.47 ± 0.06	0.19 ± 0.03	0.12 ± 0.02	0.10 ± 0.01
	Spleen	0.65 ± 0.10	3.61 ± 0.48	2.29 ± 0.36	1.80 ± 0.28	0.85 ± 0.18	0.61 ± 0.09	0.40 ± 0.07	0.19 ± 0.03	0.14 ± 0.03	0.12 ± 0.02
	Lung	0.15 ± 0.04	0.47 ± 0.12	0.75 ± 0.17	0.44 ± 0.10	0.33 ± 0.09	0.31 ± 0.08	0.20 ± 0.04	0.18 ± 0.02	0.18 ± 0.03	0.14 ± 0.01
	Kidney	1.51 ± 0.41	4.55 ± 1.29	4.67 ± 1.81	2.52 ± 1.00	0.81 ± 0.27	0.57 ± 0.27	0.10 ± 0.02	0.10 ± 0.01	0.10 ± 0.01	0.10 ± 0.01
	Hippocampus	0.46 ± 0.05	0.98 ± 0.15	1.59 ± 0.24	0.71 ± 0.09	0.41 ± 0.08	0.28 ± 0.10	0.20 ± 0.07	0.23 ± 0.09	0.19 ± 0.09	0.17 ± 0.08
	Cerebral cortex	1.01 ± 0.15	1.68 ± 0.31	1.09 ± 0.25	0.77 ± 0.12	0.41 ± 0.07	0.25 ± 0.08	0.21 ± 0.06	0.12 ± 0.02	0.12 ± 0.02	0.10 ± 0.02
	Striatum	0.34 ± 0.10	0.50 ± 0.14	1.03 ± 0.23	0.31 ± 0.06	0.28 ± 0.08	0.11 ± 0.03	0.05 ± 0.01	0.03 ± 0.01	0.03 ± 0.01	0.03 ± 0.01
